# Revised porcine IgG subclass classification based on innate immune cell activation

**DOI:** 10.3389/fimmu.2026.1838862

**Published:** 2026-07-31

**Authors:** Hans Van der Weken, Charlotte Helsmoortel, Jeroen Van Iseghem, Matthias Dierick, Bert Devriendt

**Affiliations:** 1 Laboratory of Immunology, Department of Translational Physiology, Infectiology and Public Health, Faculty of Veterinary Medicine, Ghent University, Merelbeke, Belgium; 2 Institute of Quantitative Biology, Biochemistry and Biotechnology, School of Biological Sciences, The University of Edinburgh, Edinburgh, United Kingdom

**Keywords:** classification, immune system, immunoglobulin G, in silico analysis, nomenclature, pig, ROS production

## Abstract

The current classification of porcine immunoglobulin G (IgG) subclasses relies on phylogenetic analysis of gene sequences, resulting in a nomenclature that is inapt to predict biological function. Here, we propose a revised, functionality-based nomenclature derived from a comprehensive re-analysis of Fc domain interaction motifs and *in vitro* effector function assays. We produced twelve recombinant porcine IgG subclasses and evaluated their ability to induce reactive oxygen species (ROS) production in porcine monocytes and neutrophils. Our results reveal a striking functional dichotomy: only the subclasses re-classified here as the pIgG4 family (IgG4a, IgG4b, IgG4c and IgG4d) induced a significant respiratory burst. In silico analysis suggests this unique ability is linked to a conserved Glycine-Proline (“GP”) motif in the lower hinge, which is absent in other subclasses. Furthermore, we predicted the structural determinants of complement activation, identifying a putative role for a “PAP” motif and Lysine-320 in the predicted C1q binding site. Subclasses with a K320E mutation (IgG4d, IgG5 family) or sequence deviations in the “PAP” motif displayed reduced (IgG5 family) or abolished (pIgG3) complement activity. Additionally, the ancestral pIgG3 Fc domain was confirmed to be functionally inert, a phenotype most-likely driven by a P329L mutation that disrupts the hydrophobic Fc domain/Fcγ receptor interface. These findings challenge the previous assumption that pIgG1 is a potent activator of all porcine myeloid cells and highlight that discrete amino acid motifs play a role in IgG effector functions. The proposed functional nomenclature provides a predictive framework for porcine immunology that enables a better understanding of IgG subclass diversification in health and disease.

## 1 Introduction

Antibodies play a critical role in protecting mammals against infectious diseases. While several antibody classes exist, Immunoglobulin G (IgG) is the predominant class in circulation and tissues, serving as a crucial bridge between adaptive and innate immunity. IgG is composed of two heavy and two light chains which are connected via disulfide bridges and contain both variable (VH and VL) and constant domains (CH1, CH2, CH3 and CL). The antigen-binding site (Fab) domain is responsible for pathogen neutralization, while the constant fragment (Fc) domain dictates the effector functions, primarily through two mechanisms: activation of the complement system and engagement with Fcγ receptors (FcγR). The latter are expressed on innate immune cells such as neutrophils, monocytes, and macrophages. These interactions activate immune cells to exert their effector functions, including antibody dependent cellular phagocytosis (ADCP), antibody dependent cytotoxicity (ADCC), complement dependent cellular cytotoxicity (CDCC) and complement dependent cytotoxicity (CDC). Therefore, antibodies and their receptors form an important link between the two branches of the immune system, allowing innate immune cells to interact with diverse antigens recognized by adaptive immune cells (1, 2).

Different IgG subclasses exist, resulting from gene duplications and further diversification during speciation. These IgG subclasses are identified based on sequence variation in the hinge region and discrete sites within the CH2 domain. In humans, four IgG subclasses (IgG1, IgG2, IgG3, IgG4) exist and have a different affinity towards FcγRs, complement component C1q and FcRn, ultimately leading to distinct effector functions (3). Human IgG1 and IgG3 strongly bind C1q and activating FcγRs (FcγRI, FcγRIIa, FcγRIIIa), whereas IgG2 and IgG4 are poor complement activators and have a lower FcγR affinity (4). FcRn extends IgG half-life and facilitates placental and mucosal transport. Human IgG3 has a lower affinity for FcRn, resulting in a reduced plasma half-life (3). In mice, four IgG subclasses are present (IgG1, IgG2a, IgG2b, IgG3), where IgG2a and 2b are potent activators of complement and bind strongly to activating FcγRs (FcγRI, FcγRIII, FcγRIV), while IgG1 preferentially binds the inhibitory FcγRIIb (5). A specific variant (IgG2c) is present in some mouse strains, with C57BL/6 producing IgG2c instead of IgG2a. Beyond rodents and humans, domestic species exhibit further subclass expansion. Cattle and goats have three orthologous IgG subclasses. Horses produce seven IgG subclasses with IgG1, IgG3, IgG4, IgG5, and IgG7 capable of eliciting respiratory bursts and complement activation, unlike IgG2 and IgG6 (6). Dogs have four IgG subclasses (IgG1-IgG4), with limited functional data available, but some evidence of differential FcγR and complement interactions (7). These differences reflect species‐specific gene duplications after mammalian speciation, underscoring that “same‐name” subclasses do not necessarily share effector functions across species.

Like other species, pigs have several IgG subclasses. Initial studies in European pig breeds (Landrace-Yorkshire) showed the presence of six IgG subclasses (IgG1-6) (**Table 1**) (8, 9). These subclasses have approximately the same size with only IgG3 possessing a slightly longer hinge. Except for IgG3, two allelic variants were discovered for the other subclasses, indicated by the letters a and b, bringing the total number of genes encoding the IgG heavy chain (*IGHG*) in pigs to 11. The international ImMunoGeneTics information system (IMGT) later reduced this to 8 distinct subclasses (IGHG1, IGHG2, IGHG3, IGHG4, IGHG5-1, IGHG5-2, IGHG6–1 and IGHG6-2) matching the nomenclature established by Eguchi-Ogawa et al. Later, an alternative nomenclature for the porcine IgG subclasses was proposed based on a phylogenetic analysis of the full *IGHG* sequence of different pig breeds, which classified the porcine *IGHG* genes into nine subclasses: *IGHG1*, *IGHG2a*, *IGHG2b*, *IGHG2c*, *IGHG3*, *IGHG4*, *IGHG5a*, *IGHG5b* and *IGHG5c* (**Table 1**) (10, 11). It is important to note here that not a single breed expresses all subclasses. For instance, while the *IGHG1*, *IGHG3* and *IGHG4* genes appeared to be present in all breeds, the presence of the *IGHG2* and *IGHG5* genes varied significantly. This variability in the *IGHG* gene cluster between pig breeds should not come as a surprise, as the European and Chinese boar diverged 1 million years ago and were domesticated independently 10,000 years ago (12–14).

**Table 1:** Nomenclature of the porcine IgG subclasses based on *IGHG* genes.

Zhang, 2020 (11)	Eguchi-Ogawa, 2012 (10)	Butler, 2009 (8)
IGHG3	IGHG3	IgG3
IGHG1	IGHG1	IgG1a
		IgG1b
IGHG5a	–	–
IGHG5b	IGHG5-1	IgG5b
IGHG5c	IGHG5-2	IgG5a
IGHG2a	IGHG2	IgG2a
		IgG2b
IGHG2b	IGHG4	IgG4a
		IgG4b
IGHG2c	IGHG6-1	–
IGHG4	IGHG6-2	IgG6a
		IgG6b

The classification of Eguchi-Ogawa et al. (2012) and Zhang et al. (2020) is based on genomic DNA, while Butler et al. (2009) worked with cDNA.

Both the latest gene-based nomenclature based on the work of Zhang et al. (11) and the IMGT nomenclature may however not capture functional differences driven by specific amino acid motifs in FcγR, complement and FcRn binding regions. Since these are based on full *IGHG* gene sequences, they overlook point mutations in functional regions that may alter effector functions. Therefore, we propose to revise the current phylogenetic-based classification to better reflect interactions with FcγR, complement, and FcRn. Such a functionality-based classification would thus better align with biological roles as is the case for human and mouse IgG subclasses. However, little is known about the ability of different porcine IgG subclasses to activate innate immune cells. A recent study produced recombinant porcine IgG subclasses based on the latest nomenclature and showed that IgG1, IgG2a, IgG2b, IgG2c and IgG4 bound well to different innate immune cells and mediated CDCC, ADCC and ADCP (15). The IgG5b and IgG5c subclasses showed a weak binding to immune cells and a poor functional activity. Interestingly, although strong binding of IgG3 immune complexes to monocytes and macrophages was observed, no functional activity was observed (15).

In this study, we revisited the porcine IGHG genetic analysis across breeds, focusing on the functional motifs within the Fc domain to propose an updated, functionality‐based IgG subclass nomenclature in pigs. We also investigated the effector functions of these subclasses, particularly their ability to induce ROS production, to support this functional classification and correlate specific amino acid differences within the pIgG subclasses to their observed activity.

## 2 Methods

### 2.1 Alignment and analysis of porcine IgG sequences

Porcine IgG nucleotide sequences were obtained from publicly available data (8, 10, 11, 16, 17). The used *IGHG* gene/allele names and available accession numbers are given in **Supplementary Table 1**. Complete amino acid sequences (CH1, hinge, CH2 and CH3 domains) derived from these sequences are shown in **Supplementary Figure 1** together with their functional context and sequence alignment. The different pIgG sequences were aligned using Clustal omega multiple sequence alignment (MSA) (version 1.2.4) (18).

### 2.2 Structural analysis of porcine IgGs

Molecular graphics and analyses were performed with UCSF ChimeraX (19, 20). The three-dimensional structure of the different porcine IgG subclasses was predicted using Colabfold/alphafold2 and matched to human IgG1 (P01857) (21–23).

### 2.3 Production of porcine IgGs

Plasmids containing an attB site, followed by the coding sequences for the light and heavy chains separated by a GT2A sequence, were synthesized by Genscript and cloned into a pcDNA3.1 vector with the G418 resistance gene. The variable heavy and light chains were derived from a porcine APN-specific mouse IgG (in-house; clone IMM013) (24). The porcine constant light chain was derived from protein accession number AAA03520.1, while the constant heavy chains were derived from the accession numbers highlighted in **Supplementary Table 1** (in blue).

A previously generated, zeocin-resistant CHO-K1 cell line containing a single landing pad (SLP) site for targeted DNA integration was transfected with the IgG subclass containing plasmids to allow for targeted integration into the SLP20 site. The SLP20 site is integrated in the CHO genome at a locus that has been validated for reproducible integration during transfection (the ROSA26 locus) (25). It contains an attP site, followed by the coding sequences for the BxB1 recombinase, green fluorescent protein (GFP) and the zeocine resistance gene, all separated by a 2A peptide derived from Thosea asigna virus with a glycine-serine-glycine linker (GT2A) sequence.

After transfection, stable IgG expressing cells were selected by adding 500 µg/mL G418 to the culture medium for 10 days and subsequently sorting them using a BD FACS Melody cell sorter (BD Biosciences). Doublets were excluded based on both side and forward scatter profiles. Cells that lost their GFP expression were selected as this indicates integration of the pIgG gene. After selection of successful transfectants, porcine IgG expressing CHO cells were expanded to a 400 mL suspension culture in suspension culture medium (EX-CELL ACF CHO medium (Sigma) supplemented with 500 μg/mL G418, 0.5% P/S, 5% (v/v) IgG-low fetal calf serum, 20 mM HEPES, 6 mM L-Glut). When cell density reached 2.0 x 10^6^ cells/mL, the temperature was lowered to 31 °C and an extra 2% (v/v) L-Glutamine was added for optimal protein production (Osinski, 2019). When cell viability dropped to 90%, the supernatant was collected and IgGs were further purified by affinity column chromatography using 1 mL Hi-Trap Protein A HP columns (Cytiva). Columns were equilibrated using PBS, loaded with cell culture supernatant and washed with PBS. Subsequently, the bound proteins were eluted with elution buffer (1 M L-Arginine, 1 M NaCl, pH 2.7), and neutralized with 50 µL neutralization buffer (1 M Tris-base, pH 9). After overnight dialysis in PBS using dialysis cassettes (Slide-A-Lyzer, 10 kDa; Thermo Fisher), the samples were retrieved from the cassettes and the concentration of the purified IgGs was determined by Nanodrop spectrophotometer (Denovix).

### 2.4 SDS-PAGE Coomassie staining

Samples of the purified IgG subclasses 1a, 1b, 2, 3, 4a, 4b, 4d, 5b and 6 (1 µg) were mixed with Laemmli buffer (5x) containing β-mercaptoethanol (1/20, Gibco) and heated for 10 min at 95 °C. Next, the samples were separated on a 12% SDS-PAGE for 2h at 123V. The gel was stained using Coomassie staining buffer (1 g Coomassie Brilliant Blue (Bio-rad), 16.7% acetic acid, 41.6% methanol in ddH_2_O), destained overnight in destaining buffer (7.5% (v/v) acetic acid, 15% (v/v) methanol in ddH2O) and visualized with a Chemidoc MP imaging system (Biorad).

### 2.5 Isolation of neutrophils

Porcine neutrophils were isolated from peripheral blood of healthy 12- to 18-week-old donor pigs using a density gradient centrifugation method, as described previously (26). Briefly, blood collected from the jugular vein was layered onto a discontinuous Percoll gradient (68% and 75%) and centrifuged at 500 × g for 30 min at 4 °C. The interface band containing polymorphonuclear cells was collected, diluted in 40 mL PBS-EDTA (1mM) and centrifuged at 400 × g for 10 min at 4 °C. Residual erythrocytes were removed by hypotonic lysis (150 mM NH_4_Cl; pH 7.5) for 10 min at room temperature. The resulting neutrophils were washed twice with calcium- and magnesium-free HBSS, and cell viability and concentration were determined using a counting chamber. Purity (>95%) was assessed using flow cytometry (Cytoflex, Beckman Coulter).

### 2.6 Isolation of monocytes

Porcine monocytes were isolated from peripheral blood mononuclear cells (PBMCs) via immunomagnetic positive selection as described before (27). Briefly, peripheral blood was fractionated over a Lymphoprep density gradient by centrifugation at 1200 x g for 10 min at 18 °C. The PBMC layer was collected, washed in PBS-EDTA (1 mM), and centrifuged at 400 x g for 10 min at 4 °C. Following erythrocyte lysis, the cell suspension was passed through a 70 μm cell strainer to remove clumps. PBMCs were incubated with an anti-CD14 monoclonal antibody (in-house; clone Mil-2) in MACS buffer (PBS-EDTA + 1% FCS) for 20 min at 4 °C, followed by incubation with goat anti-mouse IgG microbeads (Miltenyi Biotec). CD14+ monocytes were separated from the CD14- fraction using LS columns on a magnetic separator (Miltenyi Biotec). The isolated monocytes were then washed twice with calcium- and magnesium-free HBSS, after which the purity of the CD14+ fraction (>95%) was confirmed using flow cytometry (Cytoflex). Cell viability and concentration were measured using a counting chamber.

### 2.7 Production of reactive oxygen species by neutrophils and monocytes

The day prior to neutrophil or monocyte isolation, a clear-bottom 96-well plate was coated with 10 μg/mL porcine APN (L6007; Sigma-Aldrich) in sterile PBS. After overnight incubation at 4 °C, the wells were blocked for 2 h at 37 °C using sterile filtered PBS + 3% (w/v) BSA solution. Next, the wells were incubated with the porcine IgG subclasses at concentrations ranging from 0 to 16 μg/mL for 2 h at 37 °C. CD14+ monocytes (2.0 x 10^5^) or neutrophils (2.0 x 10^5^) were added to each well, along with 125 μL 0.05 mM luminol diluted in HBSS (with Ca2^+^ and Mg2^+^). Phorbol 12-myristate 13-acetate (PMA) was used as a positive control. All conditions were tested in duplicate. Using a Luminoskan (Labsystems), background chemiluminescence was first measured in relative light units (RLU) for 5 min, followed by 2h during which the chemiluminescence of the assay was measured. The ROS production was expressed as relative light units and measured as the total area under the curve (AUC) integrated over the 2-hour measurement window.

### 2.8 Assessment of APN binding functionality

To assess if the purified IgG subclasses were still able to bind to their target antigen (APN), a flow cytometry binding assay was performed using porcine APN-expressing BHK-21 cells. Here, 2.0 x 10^5^ APN-expressing BHK cells were incubated with the different IgG subclasses (5 µg/mL) for 30 min at 4 °C in staining medium (RPMI + 1% FCS). After incubation, the cells were washed in staining medium and incubated for another 30 min at 4 °C with a FITC-conjugated anti-pig IgG antibody (A100-205F; Bethyl) to validate the binding of the pIgGs to the APN-expressing cells. Binding of the pIgG subclasses was subsequently assessed with a CytoFLEX flow cytometer (Beckman Coulter).

### 2.9 Statistical analysis

To assess differences in monocyte and neutrophil ROS production after stimulation with the different IgG subclasses, a 2-way ANOVA was performed using Graphpad Prism 9. For the analysis of the IgG1a, 1b, 2, 3, 4a, 4b, 4d, 5b and 6 subclasses, 5 independent biological replicates were used by isolating immune cells from five distinct donor pigs. For the analysis of the IgG4c, 5a and 5c subclasses, 4 independent biological replicates were used from 4 different donor pigs. Normal distribution was confirmed using the Shapiro-Wilk test. The homogeneity of variances was confirmed using a homoscedasticity plot. The two-stage step-up method of Benjamini, Krieger and Yekutieli was used to correct for multiple comparisons.

## 3 Results

### 3.1 In silico sequence analysis

In humans and mice, the different subclasses were originally numbered based on their relative abundance in serum but are now categorized by their ability to trigger specific immune pathways. Therefore, interactions with specific FcγRs, FcRn and C1q binding allows to distinguish the different subclasses. For pig, these interactions are largely unknown, making their correct classification difficult.

**Figure 1:**
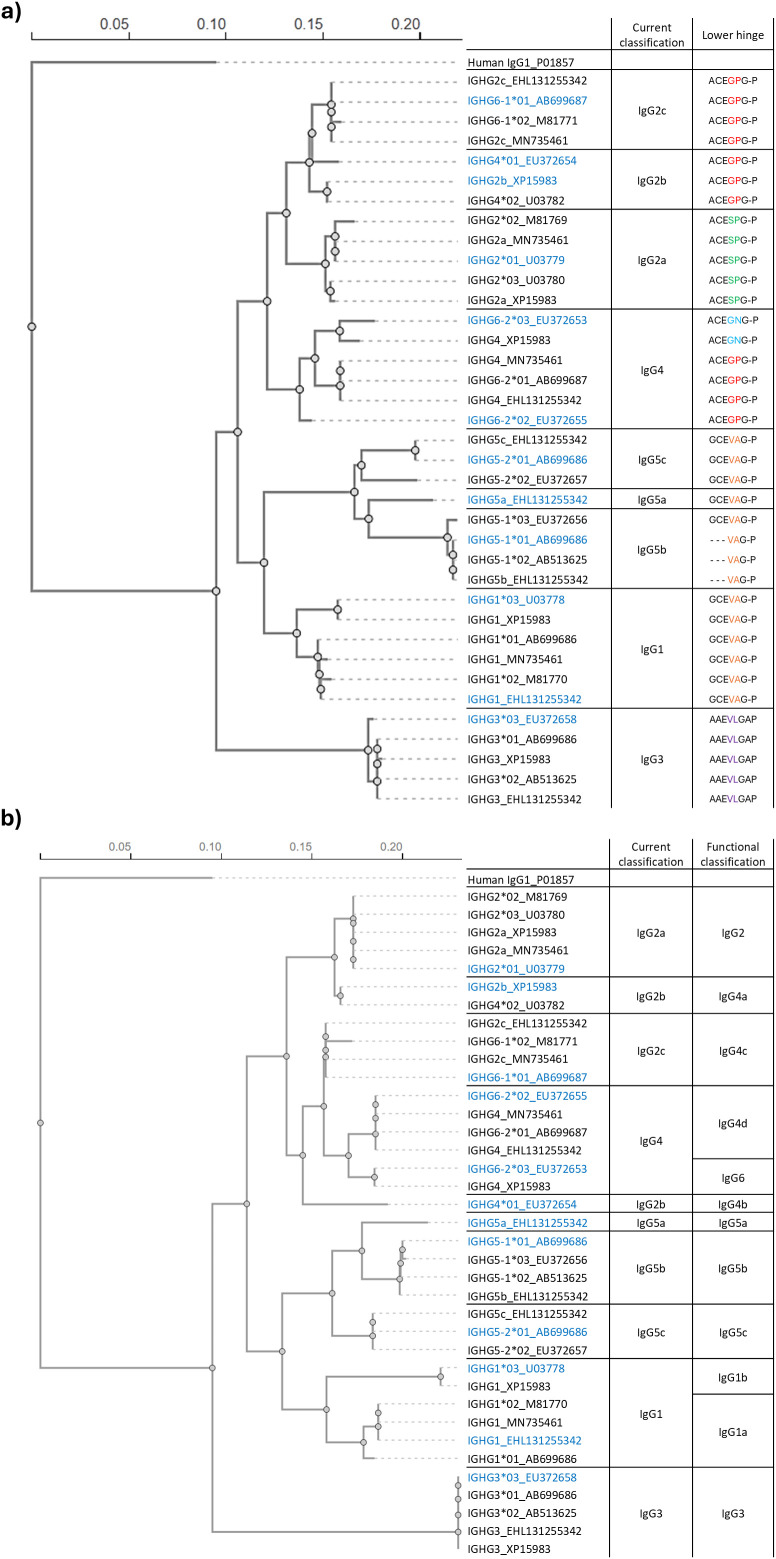
Phylogenetic trees based on **(A)** full IGHG gene sequence and **(B)** predicted interaction motifs of 37 porcine IGHG genes and their revised IgG subclass classification. The predicted interaction motifs used for the generation of the phylogenetic tree are provided in **Figure 2B**. The lower hinge sequence is provided in panel a, with the different predicted FcγR interaction motifs highlighted in different colors (“GP” in red, “SP” in green, “GN” in blue, “VA” in orange and “VL in purple). A human IgG1 sequence is used as an outgroup/reference sequence to root the phylogenetic tree. The x-axis represents the output distance matrix and is used as a measure to show sequence similarity. The porcine IgG sequences that were used to evaluate porcine IgG effector functions are indicated in blue. The current classification is based on the study from Zhang et al., 2020 (11).

**Figure 2:**
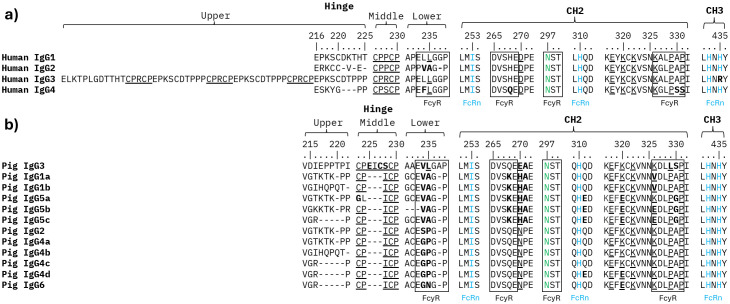
Sequence variation of key interaction sites in the IgG Fc domain for **(A)** the four human IgG subclasses and **(B)** the twelve porcine IgG subclasses. FcγR interaction sites are shown in squares, while C1q and FcRn interaction sites are underlined and denoted in blue, respectively. Key differences between the IgG subtypes are indicated in bold. The residues are numbered using the EU numbering scheme (Kabat et al., 1991) (44); this scheme is based on human IgG1 sequences and therefore does not assign numbers for most amino acids of the long IgG3 hinge sequence.

The latest nomenclature of the porcine IgG subclasses is based on a phylogenetic analysis of the full *IGHG* genes of different pig breeds (11). However, human and mice studies have revealed that single point mutations at critical locations within the hinge and Fc domain sequence can alter IgG functionality. Hence, we performed a thorough re-analysis of 37 available porcine *IGHG* genes derived from different pig breeds, focusing on predicted key regions involved in FcγR, FcRn and C1q binding. Here, an important mismatch between the current IgG subclass nomenclature and a previously predicted FcγR binding site (**Figure 1A**) was observed (8). Specifically, sequences within the IgG4 subclass showed two sets of FcγR binding motifs (“GP” and “GN”) in the lower hinge at positions 234 and 235. Since this site is critical for FcγR interaction and a single subclass cannot have two distinct functions, we propose a new and updated, functionality-based nomenclature of the pig IgG subclasses (**Figure 1B**). Therefore, we clustered the same 37 available porcine IgG genes into 12 distinct IgG subclasses based on their hinge region (upper, middle, lower) and predicted FcγR, FcRn and C1q interaction motifs (**Figure 2**). From here on, we will use this revised nomenclature, with the current nomenclature in parentheses if it differs and is mentioned for the first time.

Based on key residues involved in FcγR, C1q and FcRn interaction, different clusters of IgG subclasses can be differentiated (**Figures 1B**, **2B**). For the predicted FcγR interaction site at positions 234 and 235, there are 5 distinct variations. The porcine subclass IgG3 has a “VL” motif, while the pIgG1 and pIgG5 subclass families have a “VA” motif, similar to human IgG2. The revised pIgG4 subclass family (formerly IgG2b,c and IgG4) has a “GP” motif, while the pIgG2 (formerly IgG2a) and pIgG6 (also formerly IgG4) subclasses have an “SP” and “GN” motif, respectively. The pIgG1 and pIgG5 subclass families also have a Lysine at position 268 and a Histidine at position 270, which is a critical residue for complement activation in humans (28). The pIgG2, 4 and 6 subclasses on the other hand have an Asparagine at position 270. Porcine IgG3 has a glutamic acid, which resembles the negative charge of aspartic acid found in humans and mice. The porcine IgG1, IgG2, IgG4 and IgG6 subclasses all retained the “PAP” motif at positions 329-331, while pIgG5 has an A330G mutation at this site. The porcine IgG3 subclass has a P329L and A330S double mutation at this critical site, which is modeled to disrupt both FcγR interaction and C1q complement activation. A K320E mutation is present in the pIgG5b/c, pIgG4d and pIgG6 subclasses (pIgG4d and pIgG6 were previously combined in the pIgG4 subclass). Porcine IgG1 possesses a K326V mutation, while pIgG5b and pIgG5c have a K326E variant at this site.

The hinge shows the highest variability among the different pIgG subclasses, with some having slightly shorter hinges (IgG5c, IgG4c, IgG4d, IgG6) and the pIgG3 subclass showing a slightly longer hinge, although not remotely comparable to the long hinge of human IgG3. Within the porcine IgG subclasses, the IgG1a/b, IgG4a/b/c and IgG5b/c subclasses only differ in their upper hinge and might not show functional differences besides different binding kinetics to their cognate antigen. Interestingly, the pIgG5a subclass has a critical C223G and P224L double mutation in the middle hinge, which would result in the loss of a disulfide bridge. This subclass is so far only detected in the Chinese Erhulian breed.

### 3.2 Evaluation of porcine IgG subclass effector functions

**Figure 3:**
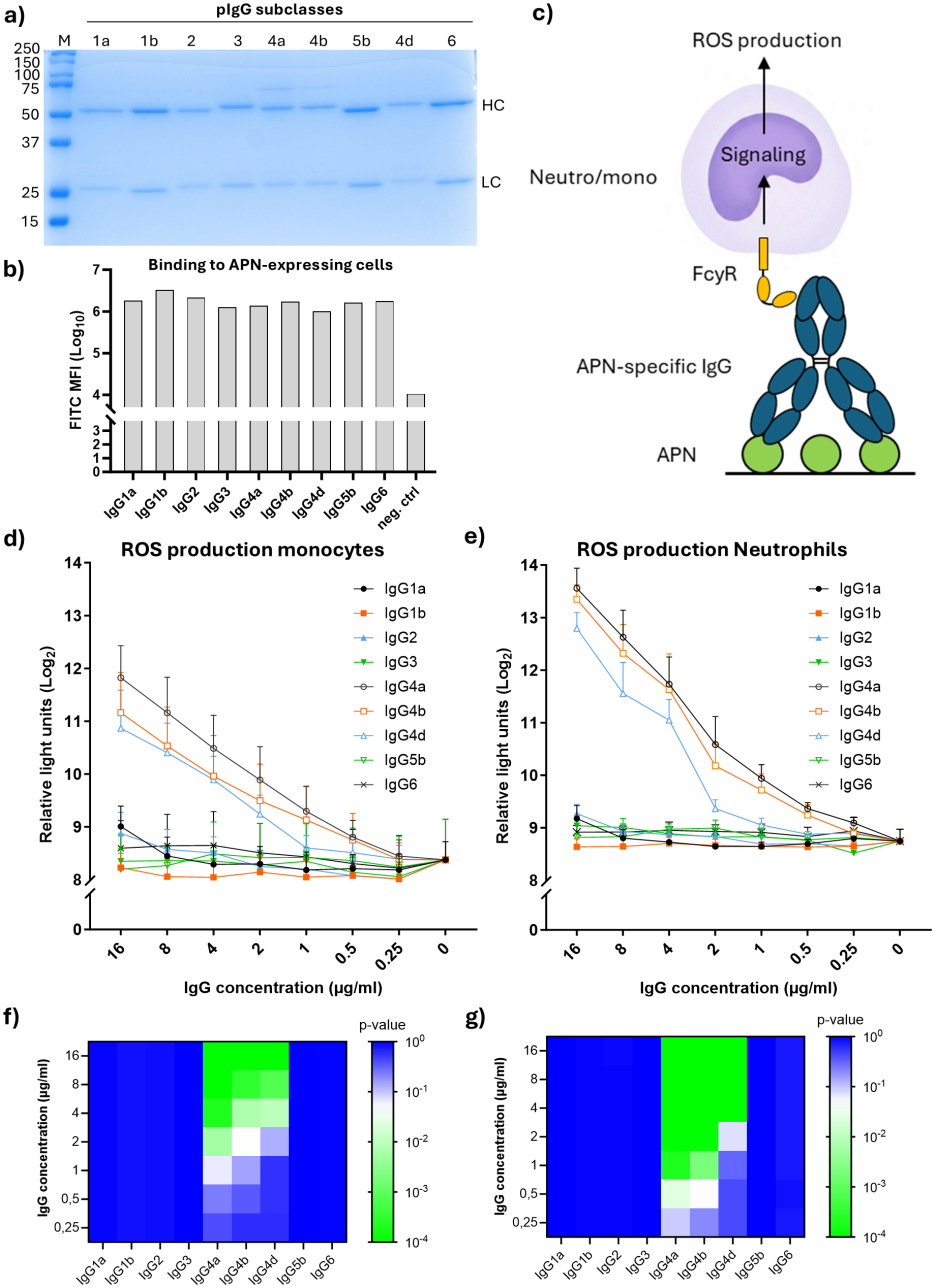
Quality assessment and functional analysis of produced APN-specific pIgG subclasses. IgG quality control by **(A)** SDS-PAGE Coomassie staining and **(B)** flow cytometry binding assay to porcine APN of 9 purified IgG subclasses with their respective mean fluorescent intensity (MFI). **(C)** Schematic overview of the ROS-assay and the Relative Light Units (RLU) produced by **(D)** monocytes and **(E)** neutrophils after incubation with different porcine IgG subclasses at different concentrations. The heatmaps represent the p-values of the **(F)** monocyte and **(G)** neutrophil ROS production for the different IgG isotypes at different concentrations. n = 5. Data points represent the mean with standard error of mean (SEM). Blue: p>0.05; white: p=0.05; green: p<0.05. M, molecular weight marker; HC, heavy chain; LC, light chain.

To determine whether the different pIgG subclasses have a varying capacity to activate innate immune cells, a selection of 9 different APN-specific IgG subclasses was made based on their sequence diversity at key interaction sites, according to the revised functionality-based nomenclature (IgG1a, IgG1b, IgG2, IgG3, IgG4a, IgG4b, IgG4d, IgG5b and IgG6). The selected IgG subclasses were produced and purified with correct predicted sizes for their heavy and light chains (**Figure 3A**) and were able to bind their cognate antigen in a flow cytometry binding assay using APN-expressing BHK-21 cells (**Figure 3B**).

Next, we examined whether these pIgG subclasses differed in their ability to induce reactive oxygen species (ROS) production by monocytes and neutrophils, which is a direct consequence of their FcγR engagement. Stimulating both monocytes and neutrophils with antigen-bound pIgG4a, pIgG4b and pIgG4d significantly increased ROS production compared to unstimulated cells, while no significant increase in ROS production was observed for pIgG1a, pIgG1b, pIgG2, pIgG3, pIgG5b and pIgG6 stimulated cells (**Figures 3C–G**).

Since these data imply that the “GP” motif found at positions 234 and 235 in the pIgG4 subclass family appears critical for the stimulation of ROS production in monocytes and neutrophils, we further assessed the remaining IgG subclasses IgG4c, IgG5a and IgG5c. These IgG subclasses were produced and purified and were able to bind to their cognate antigen in a flow cytometry assay (**Supplementary Figure 2A**). Next, their ability to induce ROS production in monocytes and neutrophils was assessed. As expected, only the pIgG4c subclass was able to increase ROS production compared to unstimulated cells, while the pIgG5a and pIgG5c subclasses did not (**Supplementary Figures 2B, C**). These results highlight the critical importance of the “GP” motif in the predicted FcγR binding site of the lower hinge.

## 4 Discussion

The classification of immunoglobulin G subclasses within species is a complex endeavor, often relying on phylogenetic analysis of full *IGHG* gene sequences. While this elucidates evolutionary lineage, it frequently masks functional disparities driven by discrete amino acid motifs in the Fc domain. In swine, the current nomenclature is based on the phylogenetic analysis of full *IGHG* genes from different breeds, and includes nine different pIgG subclasses (11). While this brought order to a complex landscape of IGHG genes that have been renamed several times over the last decades (**Table 1**), our findings strongly suggest that a nomenclature rooted in biological function, specifically the ability to engage Fcγ receptors (FcγR), provides a more accurate and predictive framework for understanding porcine humoral immunity. Therefore, we propose a shift towards a function-based nomenclature for porcine IgG, validated by profiling of effector functions and *in silico* sequence analysis. Our data reveal that specific motifs in the hinge and CH2 domain, conserved across species or uniquely divergent, are robust predictors of innate immune activation. A core understanding of these motifs is essential for predicting IgG subclass effector function.

The primary binding interface for FcγRs in human IgG is located within the lower hinge and the proximal CH2 domain, located at positions 233-239, 265-269, 327–331 and a glycosylated asparagine at position 297 (3). Together, these form the common binding interface essential for interaction with all FcγRs. Critical residues in human and mouse IgG within this binding interface include L234, L235, G236, P238, D265, N297 and P329. Our data show that pIgG1, containing a “VA” lower hinge motif at position 234 and 235, does not induce ROS production by monocytes and neutrophils. However, Paudyal et al. (2022) reported that pIgG1 mediates strong Antibody-Dependent Cellular Phagocytosis (ADCP) in macrophages (**Table 2**). This discrepancy is likely driven by a differential receptor expression on the effector cells used. In pigs, neutrophils and monocytes express high levels of FcγRIIIa and FcγRIIa/b, but lower levels of the high-affinity FcγRI compared to macrophages (29, 30). The “VA” motif in pIgG1 mimics the lower hinge sequence of human IgG2, which exhibits a low affinity for the activating receptor FcγRIIIa but retains binding to the inhibitory FcγRIIb and the high-affinity FcγRI (31). We hypothesize that porcine monocytes and neutrophils, which rely heavily on FcγRIIIa for oxidative burst induction, fail to activate in response to pIgG1 due to this reduced affinity threshold, whereas macrophages expressing high levels of FcγRI remain competent for ADCP.

**Table 2:** Functional differences between different pIgG subclasses.

Functional classification	Zhang, 2020 (11)	Binding to immune cells				Fc effector functions			
		Monocytes	Neutrophils	NK cells	CDCC		CD107a degranulation	ADCP	ROS production
IgG3	IgG3	+	++	+/-	–		–	–	–
IgG1a	IgG1	++	+++	+++	+++		+++	+++	–
IgG1b	/	(++)	(+++)	(+++)	(+++)		(+++)	(+++)	–
IgG5a	IgG5a	(+/-)	(+)	(+)	(–)		(+)	(++)	–
IgG5b	IgG5b	+/-	+	+	–		+	++	–
IgG5c	IgG5c	+/-	+	+	+		+	++	–
IgG2	IgG2a	++	–	++	+++		+++	+++	–
IgG4a	IgG2b	+++	–	++	+++		+++	+++	+++
IgG4b	/	(+++)	(–)	(++)	(+++)		(+++)	(+++)	+++
IgG4c	IgG2c	+++	–	++	+++		+++	+++	+++
IgG4d	IgG4	+/-	++	++	++		+++	+++	++
IgG6	/	(+/-)/(++)	(–)/(++)	(++)	(++)		(+++)	(+++)	–

+++: very strong. ++: Strong. +: average. +/-: Weak. -: not present/negligible. Values in brackets are predicted based on sequence similarity. pIgG subclass order was determined based on sequence similarity and functionality, placing similar subclasses closer together. Binding data to immune effector cells (monocytes, macrophages and NK cells) and Fc effector functions for complement dependent cellular cytotoxicity (CDCC), CD107a degranulation and antibody dependent cellular phagocytosis (ADCP) was derived from Paudyal et al., 2022 (15).

A key finding in our study is the functional segregation of the pIgG4 subclass family (IgG4a, IgG4b, IgG4c, IgG4d), defined by a unique Glycine-Proline (“GP”) lower hinge motif at positions 234 and 235. This family was the sole inducer of respiratory burst activity in neutrophils and monocytes (**Figure 3**; **Supplementary Figure 2**) and identifies the “GP” motif as a predictive marker for myeloid activation in swine, analogous to the “LLGG” motif in human IgG1 and IgG3. In humans, L324 and L235 in IgG1 and IgG3 are critical for high-affinity binding to FcγRIIIa and FcγRIIa (32, 33). The porcine “GP” motif appears to function as the structural equivalent, likely stabilizing the lower hinge conformation necessary for engaging the activating FcγRs (likely FcγRIIIa) abundant on porcine neutrophils and monocytes. Conversely, the “SP” and “GN” motifs found in pIgG2 and pIgG6, respectively, failed to trigger ROS production. This suggests that while these subclasses may bind FcγRs, as indicated by strong ADCP and NK cell activation (15), they fail to induce the necessary receptor clustering or cross-linking required for the oxidative burst, or preferentially engage inhibitory receptors (FcγRIIb). Alternatively, it might be the “GP” motif in the pIgG4 subclass family that fails to bind to FcγRIIb, preventing dampening of the ROS production as opposed to other pIgG subclasses. It is important to note here that within the key interaction domains put forward in our sequence analysis, both IgG2 (G234S) and IgG6 (P235N) only differ at a single amino acid compared to IgG4a and IgG4d respectively, which further reenforces the idea that the “GP” motif at positions 234–235 plays a key role in FcγR binding. Further experimental validation is required to definitively resolve these mechanistic possibilities by assessing binding kinetics and determine the precise affinities of the different porcine IgG subclasses towards individual FcγRs. Another critical residue involved in FcγR binding is a proline at position 329, which packs against tryptophan residues in FcγRs to form a critical hydrophobic sandwich. In humans, a P329G mutation at this site completely silences its effector function (34, 35). Porcine IgG3 has a similar P329L mutation, which might prevent its binding to FcγRs, explaining why this subclass does not appear to activate any immune cells and is the only subclass that fails to activate NK cells (15). Ultimately, both mutational studies and further elucidation of the affinities of the porcine IgG subclasses for different FcγRs will be essential in validating these sequence-based predictions.

**Figure 4:**
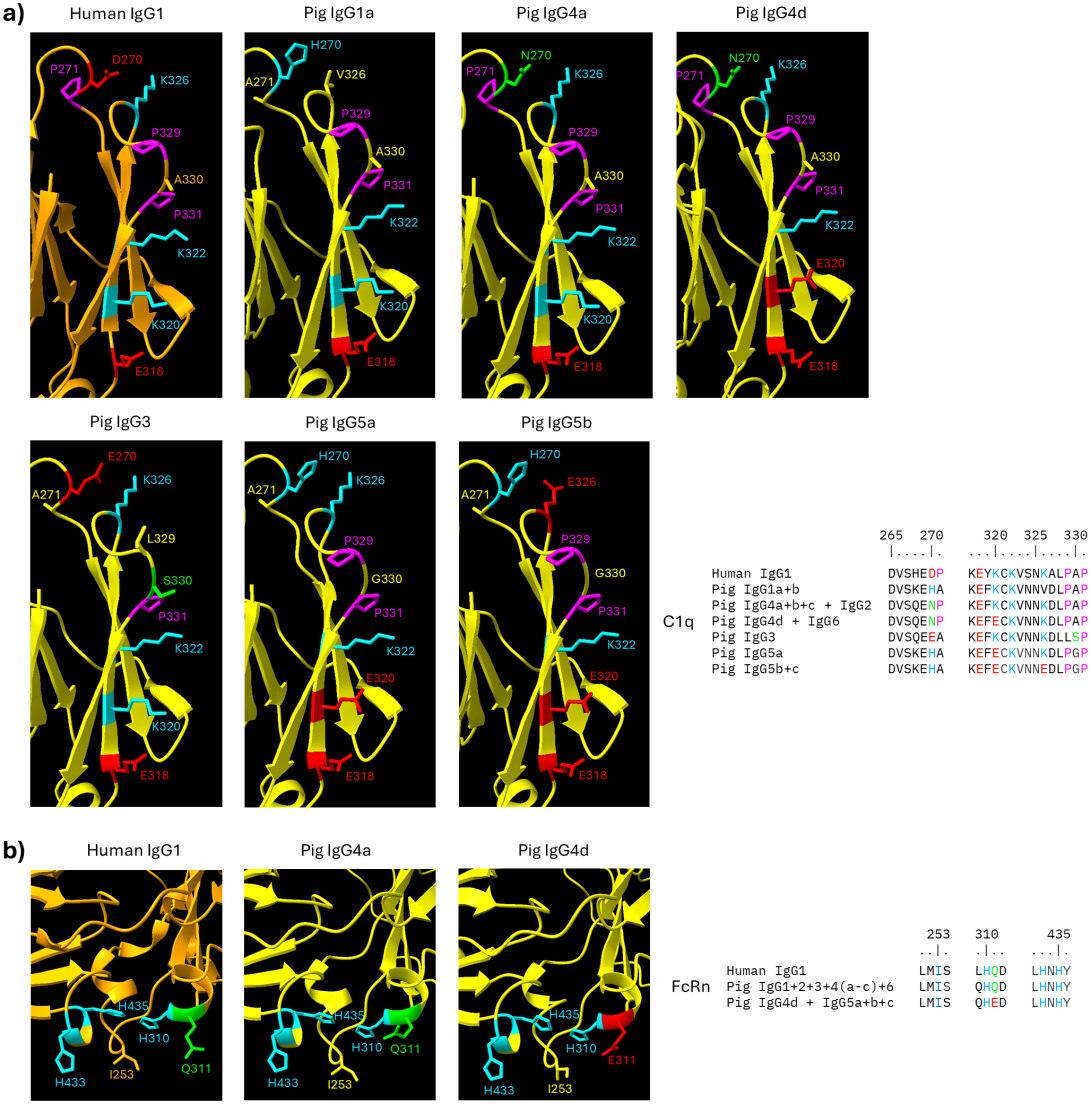
Structural variation of different pIgG subclasses. Key amino acid residues involved in **(A)** C1q and **(B)** FcRn binding are highlighted. Red: negative charge (D/E); blue: positive charge (K/H); green: polar (S/N/Q); yellow/orange: hydrophobic (G/A/V/L/I); purple: proline.

Complement activation is mediated by the C1q component binding. In human and mouse IgG, key residues involved in C1q interaction are located in the CH2 domain, particularly D270, K322, P329 and P331 (**Figure 4A**) (28, 33). By combining our sequence analysis with the *in vitro* experiments on CDCC (15), we identified a similar Proline-Alanine-Proline (PAP) motif at positions 329–331 as a potential determinant for complement activation (observed in the porcine IgG1, IgG2, IgG4 and IgG6 subclass families). This is identical to the “PAP” motif in human IgG1-3, which are also potent complement activators. In contrast, human IgG4 features a “PSS” motif at the C1q binding site, which attenuates its complement activation. In pigs, a “PGP” motif is found in the weak complement activating IgG5 subclasses, while an “LSP” motif is found for pIgG3, which is unable to activate the complement system (**Table 2**). These subtle differences underpin the observed variability in complement activation between the porcine IgG subclasses and might explain why the ancestral pIgG3 subclass appears to be functionally silent. One could speculate why such a non-effector IgG subclass would be evolutionary retained. One hypothesis would be that the porcine IgG3 serves a non-inflammatory role, acting as a decoy to clear antigens without triggering damaging inflammation or playing a role in B-cell regulation independent of effector function. However, other residues can also play a role in the C1q interaction. Although the critical K322 is conserved within all pIgG subclasses, the strong complement activators (IgG1, IgG2 and IgG4a-c) possess a lysine at position 320, while the slightly weaker complement activator IgG4d (and IgG6) and the non-activating pIgG5 subclasses possess a glutamic acid (E) at this position (**Table 2**; **Figure 4A**). Complement activation for the pIgG6 subclass has not been assessed yet but based on sequence similarity, it is predicted to be similar to pIgG4d. Both E318, K320 and K322 have been implicated to be important for C1q binding in mouse studies, with the conserved K322 playing a critical role (36). The substitution of a positively charged lysine (K320) with a negatively charged glutamic acid (E320) potentially reduces the electrostatic interaction required for C1q docking, offering a structural hypothesis as for why pIgG4d has been noted to exhibit reduced complement activity compared to the other IgG4 subclasses, despite sharing a similar “PAP” motif. Besides this K320E mutation, the pIgG5 subclasses also contain a “PGP” motif (A330G mutation) alongside E320. The combination of the conformational change induced by glycine and the charge repulsion from glutamic acid possibly renders pIgG5 a poor complement activator. Another interesting difference is K326 present in the pIgG2, -3, -4 and -6 subclasses, which is substituted for a hydrophobic valine in pIgG1 and a negatively charged glutamic acid in the pIgG5b and -c subclasses (**Figure 4A**). A previous study in humans indicated that the charged lysine residue at position 326 might impair the interaction with C1q (37). Furthermore, a double mutation (E333S/K326W) increased the ability of C1q to bind human IgG1 and allowed a non-reactive IgG2 to bind C1q (38). Since the pIgG1 subclass has a non-polar valine at this position, this might result in stronger complement activation. Interestingly, the K326W mutation in human IgG1 also debilitated ADCC activity, implying that K326 also plays a role in FcγR interaction and might play a role in the inability of the porcine IgG1 subclass to induce ROS production in monocytes and neutrophils.

A last difference between the porcine IgG subclasses is located at position 270 and 271. In humans and mice, D270 is located in the BC loop (residues 266-272), is followed by a proline and plays a critical role in C1q binding (28, 33). Porcine IgG subclasses do not possess similar amino acids but rather have three different variants. The pIgG2/4/6 subclasses are quite similar, showing only a D270N mutation. On the other hand, porcine IgG3 (D270E + P271A) and pIgG1 and 5 (D270H + P271A) have more critical mutations compared to what is found in human and mouse, with IgG1 and 5 having an additional Q268K mutation. The implications of this are unclear, since these variations have not been observed in human or mouse IgG subclasses, but due to their critical location might play key roles in both C1q and FcγR interactions, especially for the pIgG1, 3 and 5 subclasses. Further research should elucidate this.

The interaction of FcRn with human and mouse IgG subclasses involves key residues I253, H310, H433 and H435 (39, 40). Human IgG3 has an arginine at position 435 and results in a lower half-life time compared to the other subclasses. Certain human IgG3 haplotypes have a R435H mutation, which increases their half-life similar to the half-life of human IgG1 (41). While all the key residues involved in FcRn interaction of human and mouse IgG are conserved in pig IgG and its subclasses, a Q311E mutation can be observed in pIgG4d and the pIgG5 subclass family (**Figure 4B**). In humans, Q311 is located at the CH2-CH3 interface, adjacent to the FcRn binding site. Replacing a polar glutamine with a negatively charged glutamic acid could alter the electrostatic environment, potentially modulating the off-rate at physiological pH. This suggests that pIgG4d and the pIgG5 family might exhibit altered pharmacokinetics, a hypothesis that warrants further investigation.

It is important to note that the functional implications of all sequence variations are not yet fully understood. For example, the allotypic variants of porcine IgG1a/b, IgG4a/b/c and IgG5b/c subclasses only differ in the upper hinge and not in critical binding domains. While the upper hinge is not directly part of the FcγR binding interface, it may modulate binding indirectly. Studies engineering the upper hinge of human IgG’s to enhance binding affinity to FcγRIIIa (CD16a) suggest that modifications here can affect the overall conformation and flexibility, potentially influencing accessibility to the binding site (32, 42). However, direct contact with FcγR appears limited to the lower hinge and adjacent CH2 domain regions, as evidenced by crystallographic data (43). This distinction is relevant when comparing allelic variants. However, the effect of sequence variations in the upper hinge on their functionality remains to be elucidated. If no functional differences can be detected, one could argue a case to merge these subclasses, reducing the total number of porcine IgG subclasses from 12 to 8.

An important caveat of this study is our use of a CHO expression system to generate the recombinant porcine IgG subclasses. While CHO cells are a robust platform for monoclonal production, their N-glycosylation profiles, specifically regarding terminal galactosylation and sialylation, differ from native porcine-derived cells. Because Fc domain glycosylation can modulate both FcγR binding kinetics and complement activation, it is possible that the absolute magnitudes of the cellular responses observed here carry an expression-system bias. While the clear-cut functional dichotomies observed (e.g., the pIgG4 family’s exclusive induction of ROS) point to a dominant role for primary amino acid motifs, future comparative studies utilizing native porcine-expressed antibodies will be highly valuable to fully clarify native physiological kinetics.

This study indicates that porcine IgG subclasses exhibit functional differences, which might be driven by specific amino acid motifs in the Fc domain, necessitating a revision of the current nomenclature. We identified the lower hinge “GP” motif, present exclusively in IgG4 subclass family, as a key driver of ROS production in myeloid cells. Furthermore, we emphasize a conserved “PAP” motif at positions 329–331 as involved in complement activation by pig IgGs by combining *in silico* sequence analysis with previously reported functional assays and C1q binding analysis. Collectively, these findings provide evidence that porcine IgG subclasses differ markedly in their ability to trigger immune effector functions, and that these differences align with distinct amino acid motifs in the Fc domain. On this basis, we propose a revised nomenclature from the gene-based nomenclature of the IMGT system and the nomenclature as proposed by Zhang et al. (11). Here, specifically the IGHG4 and IGHG6–2 genes warrant reclassification, since these subclasses appear to harbor distinct variations at positions 234 and 235, which appear to be critical in activating porcine innate immune cells. By focusing on effector function, a more accurate and meaningful understanding of antibody-mediated immunity in pigs can be achieved. While this study proposes a functional taxonomy, further biophysical validation is required to confirm the molecular mechanisms underlying these subclass-specific activities. Future studies should therefore determine the dissociation constants (KD) of these subclasses for individual porcine Fcγ receptors (FcγRI, FcγRIIa/b, FcγRIIIa), FcRn, and C1q. Complementary mutational analyses targeting the identified motifs are also required to validate the contribution of specific amino acid residues to effector function.

## Data Availability

The datasets presented in this study can be found in online repositories. The names of the repository/repositories and accession number(s) can be found in the article/**Supplementary Material**.

## Supplementary material

Supplementary Figure 1 Sequences for the different human IgG (1, 2, 3 and 4), mouse IgG (1, 2a,b,c and 3) and pig IgG (1a,b, 2, 3, 4a,b,c,d, 5a,b,c and 6) with the **(A)** CH1, **(B)** hinge, **(C)** CH2 and **(D)** CH3 domains, with predicted binding regions for FcγR shown in brackets, FcRn in blue and C1q underlined. The residues are numbered using the EU numbering scheme (Kabat et al., 1991) (44); this scheme is based on human IgG1 sequences and therefore does not assign numbers for most amino acids of the long IgG3 hinge sequence. Pig IgG shown in blue were produced and used to perform the functional assays.

Supplementary Figure 2 **(A)** Flow cytometry binding assay to porcine APN of 3 purified IgG subclasses (4c, 5a and 5c) with their respective mean fluorescent intensity (MFI) and ROS-assay with the Relative Light Units (RLU) produced by **(B)** monocytes and **(C)** neutrophils after incubation with the porcine IgG subclasses 4c, 5a and 5c at different concentrations. n = 4. Data points represent the mean with Standard error of mean (SEM). *p<0.05; ****p<0.0001.

Supplementary Table 1 Porcine IgG sequences and accession numbers. The porcine IgG sequences that were used to evaluate porcine IgG effector functions are indicated in blue.
